# Kerosene Fatalities

**Published:** 1869-02

**Authors:** 


### KEROSENE FATALITIES.

Kerosene is so cheap and makes such a brilliant light, that it will continue to be burned for illuminating purposes until all the " coal oil " is used up. We would not allow one of the dangerous detonators to be brought within the walls of our " castle;" one method of being " blown up " is quite enough for us; but the most beautiful light we ever saw for domestic use, where gas is not manufactured to hand, was exhibited recently: it was the unexplosive kerosene burning without any chimney or other machinery. It seemed to us to be one of the simplest devices ever offered to public patronage; the light is intense, clear, brilliant; no smoke, no smell. We give it as our impression that active and enterprising young men could make money handsomely by attaining the exclusive privilege of selling these lamps in specified districts, without any other capital than enough to purchase half a dozen at a time; these lamps are made of tin or glass, and can be carried about rapidly all over the house or hung against the wall.

